# Hydroxypropyl Methylcellulose Orodispersible Film Containing Desloratadine for Geriatric Use: Formulation and Evaluation

**DOI:** 10.2174/1871523022666230816090942

**Published:** 2023-09-18

**Authors:** Aya Yahya Fayez Al-Oran, Evrim Yenilmez

**Affiliations:** 1 Department of Pharmaceutical Technology, Anadolu University, Graduate School of Health Sciences, Anadolu University, Eskişehir, Turkey;; 2 Department of Pharmaceutical Technology, Faculty of Pharmacy, Anadolu University, Eskişehir, Turkey

**Keywords:** Orodispersible film, desloratadine, oral strip, oral film, allergic symptoms, geriatric

## Abstract

**Background:**

Oral strip is very similar to thin strip of postage stamp in shape, size and thickness. The strip is designed to be placed on the tongue or any oral mucosal tissue which immediately gets wet and hydrated after being in contact with the saliva. Desloratadine is one of the better-known second-generation antihistamines that has been studied for being effective in relieving the allergic nasal and skin symptoms.

**Objective:**

The aim of this study is to develop desloratadine orodispersible film (ODF) with fast disintegration time and suitable mechanical strength to treat allergic symptoms in geriatric patients in order to increase compliance and convenience.

**Methods:**

Solvent casting method using hydroxypropyl methylcellulose (HPMC) as the film forming polymer was applied. Polyethylene glycol 400 (PEG 400) and glycerol (Gly) were used as the plasticizers and citric acid (CA) was used as saliva stimulating agent. The resultant films were evaluated for disintegration time, folding endurance, surface pH, weight variation, thickness, surface morphology using scanning electron microscopy, drug content, content uniformity, moisture loss, moisture uptake, and drug-excipient compatibility using DSC and FT-IR.

**Results:**

All the selected films started to disintegrate in less than 14 seconds. Selected optimum films exhibited good mechanical properties with a folding endurance value greater than 100. The uniformity in weight, thickness, and drug content in the selected films was obtained. Surface pH was within the normal range (6.4 - 6.8). A smooth surface of the films was obtained and drug-excipient compatibility was proved using DSC and FT-IR. The dissolution test was done for optimum film formulations by simulating the oral cavity physiological conditions using the conventional dissolution test apparatus. More than 87% of the drug was released by the 4th minute.

**Conclusion:**

Orodispersible film of desloratadine was successfully prepared by solvent casting method in order to improve the disintegration/dissolution of the drug in oral cavity and hence better patient compliance and effective therapy.

## 1 INTRODUCTION

The oral route is the most patient-compliant and conventional route of drug administration, therefore about 60% of total dosage forms are administered orally. Among all the pharmaceutical dosage forms delivered orally, solid dosage forms are the most commonly used medications to obtain the desired therapeutic outcomes due to ease of administration [1, 2].

Elderly, that represents 16% of the total population, consume 31% of the medications which makes them the major consumers of medicines. Moreover, it’s believed that the per- centage of elderly is increasing significantly within the next 30 years in both developed and developing countries. The swallowing difficulties are related to age, disease related swallowing impairment (dysphagia), or simply due to polypharmacy in which the patient has to take several medications within the day to treat multiple conditions [3, 4]. Accordingly, the development of oral disintegrating tablets (ODTs) has taken increased attention among researchers and pharmaceutical companies over the last few years. ODTs are defined as a solid dosage form containing medicinal substances that disintegrate within seconds upon introduction on the tongue, as a consequence, there is no need to chew the tablet, swallow an intact tablet, or swallow the tablet with water [5, 6]. However, the continuous development of oral dosage forms has led to the convention of oral strip technology as an alternative to ODTs and to overcome their disadvantages. The main drawback of ODTs is that they are fragile and breakable, which needs special requirements related to packaging during storage and transportation [7]. Moreover, special equipment’s are needed to manufacture them [5, 8].

Oral strip is very similar to a thin strip of postage stamp in shape, size and thickness. The strip is designed to be placed on the tongue or any oral mucosal tissue and immediately gets wet and hydrated after being in contact with the saliva. The thin strip then rapidly begins to disintegrate and dissolve to release the medication. In the literature, several terms can be found to describe this technology, such as oral film or strip, thin strip, orally dissolving film, orodispersible film (ODF), buccal film, mucoadhesive film, transmucosal film, flash release wafer, quick dissolve film, disintegrating film, melting film and melt-away film [9-13]. The last two terms are not preferably used by the Food and Drug Administration (FDA) because they indicate the melting of the film instead of what actually happens; the film does not melt but disintegrates and dissolves. Instead “soluble film” or “oral soluble film” are commonly used by the FDA to avoid any misunderstanding [10]. The European Medicines Agency (EMA) prefers to use “orodispersible film” to describe the fast dissolving films, not to confuse it with “buccal film” which is designed to stay longer on the mucosa [12]. Furthermore, the European Pharmacopoeia (Ph. Eur.) 7.4 included the “orodispersible film” as a subchapter of “Oromucosal Preparations” whereas the mucoadhesive buccal films are included in the “Mucoadhesive preparations” and defined as “single-or multi-layer sheets that adhere to the buccal mucosa and may dissolve” [14].

Allergies, including allergic rhinitis and urticaria, are very common high prevalence rate disorders that have a tremendous effect on the quality of life. Early diagnosis and treatment of allergic diseases using a medication of a high safety profile are of prime importance [15]. Histamine plays a main role in the development of allergy symptoms by the activation of H1 receptors. These symptoms include sneezing, rhinorrhea, mucosal edema, as well as swelling, pruritus, and redness of the skin. Histamine blockers are essential medications for the treatment of allergic diseases. Desloratadine (DSL) is one of the better-known second generation antihistamines that has been studied for being effective in relieving allergic nasal and skin symptoms. It has a long-acting and non-sedative effect which makes it a safe and frequently used drug in the treatment of allergic rhinitis and urticaria [16].

The aim of our study is to develop and characterize ODFs of the desired dose of DSL (5 mg) that disintegrate in a matter of seconds intended for geriatric use in order to improve their compliance, convenience, and adherence when treating allergies. Hydroxypropyl Methyl Cellulose (HPMC) was used as the film forming polymer and different plasticizers were studied: glycerin (Gly), polyethylene glycol 400 (PEG 400), and propylene glycol (PG). Citric acid (CA) was used as a saliva stimulating agent since rapid disintegration of the oral film can be enhanced by more saliva in the oral cavity.

## 2 MATERIALS AND METHODS

DSL was gifted from Berko İlaç, Turkey. HPMC, Gly, PEG 400, potassium dihydrogen phosphate (KH_2_PO_4_) were purchased from Sigma, USA. PG, anhydrous calcium chloride (CaCl_2_), ethanol, CA, methanol, acetonitrile, orthophosphoric acid (H_3_PO_4_), pH 6.8 phosphate buffer tablets were obtained from Sigma-Aldrich, Germany. The water used in all experiments was freshly collected from a Millipore water purification system (Milli Q, France).

### 2.1 Preparation of ODFs and Initial Selection of the Optimal Formulations

Solvent casting method has been used to prepare the films [17]. Different ratios of HPMC and plasticizers have been studied. Different polymers and plasticizers with different ratios have been studied in order to choose the formula with the best film forming properties and high transparency [18]. In the formulation trials, mixtures with different polymer ratios were prepared, and the most suitable polymer mixtures were determined in terms of mechanical properties and transparency, and studies were carried out to obtain the optimum formulation. The composition of the formulations is presented in Table **1**. The mechanical properties of the film depend on the type of polymer and the amount of polymer used.

Required amount of HPMC was weighed and dispersed in the solvent mixture of ethanol and water. By the help of a magnetic stirrer (Wisd Laboratory Instruments, Daihan SMH5-3, Korea), a homogeneous viscous solution was formed. Plasticizer, CA, and calculated amounts of DSL were added to the previous solution and mixed to get a clear solution. The solution was degassed by sonicating it for 5 minutes with the help of an ultrasonic bath (Wisd Laboratory Instruments, Korea). The solution was poured into a petri dish of 8.5 cm diameter (İsolab, Turkey) and dried at 40°C for the next 24 hours inside a hot air oven (Nüve, FN 500, Turkey). The dried films were gently separated from the petri dish and cut into desired sizes (2×2 cm). Finally, the films were wrapped in aluminum foil and left inside a desiccator (İsolab, Turkey) containing CaCl_2_ at room temperature until further use.

Selecting the best formulas among all the formulations prepared for further studies was the most challenging step during this study. Initially, attention has to be paid to the physical appearance and general homogeneity of the films by visual examination and by touch [19].

### 2.2 Characterization of ODFs

#### 2.2.1 Disintegration Time

The film of 4 cm^2^ was placed in a petri dish containing 10 mL of an artificial saliva medium at room temperature (25°C ± 2°C) with swirling every 10 secs to simulate the oral conditions. The time required for the film to break was determined visually and considered as the disintegration time (n = 4) [17, 20].

#### 2.2.2 Mechanical Properties Evaluation

Manual folding endurance test was conducted to study the mechanical properties of the films. The film was folded in the same place until it started to break. The number of times before the film broke or developed, a visible crack was considered as the folding endurance value (n = 4) [21].

#### 2.2.3 Surface pH

The pH of the film was measured by allowing the film to get wet first by placing it in a petri dish containing 1 mL of distilled water for 1 minute. The pH meter electrode (Mettler Toledo, USA) was brought near the surface of the oral film and the value was recorded after equilibration (n = 3) [17, 20, 21].

#### 2.2.4 Weight Variation

10 films of each selected formula were randomly selected from different batches and their weights were recorded using a sensitive balance (Mettler Toledo, USA). % of weight variation and SD were calculated [17].

#### 2.2.5 Thickness

6 films of each selected formula were randomly selected from different batches and their thicknesses were recorded using a digital Vernier caliper (China Factory, Guangzhou, China). The thickness was measured at 5 different locations of the film; the four corners and the center [22].

#### 2.2.6 Scanning Electron Microscopy

A small piece of the film was coated with gold on carbon tape and placed on a circular aluminum stub in a high vacuum evaporator. Accelerating voltage of 15 kV was used for imaging. The morphological images of the film were obtained by scanning electron microscope (SEM) (Zeiss, Supratm 50 VP, Germany) at 3 definite magnifications; 1.2 K X, 2.5 K X, and 5.0 K X. In order to give a proper judgment, 2 films of the excluded ones because of their visible roughness on one side were subjected to SEM test at 200 X, 500 X and 1.2 K X magnifications to provide an indicator of smoothness/roughness for evaluating the selected films.

#### 2.2.7 Drug Content and Content Uniformity

Validated high pressure liquid chromatography (HPLC) method was used to analyze the amount of DSL in the films using HPLC apparatus (Shimadzu, LC 20-AT, Japan) equipped with a diode array detector set at 247 nm using LiChrospher 100 RP-18 octadecyl silane column (5 μm, 150×4.6 mm) (Merck, Millipore, Germany). The mobile phase was a mixture of pH 3 phosphate buffer/acetonitrile/ methanol (50:40:10, v/v/v). The flow rate and the injection volume were 0.8 mL/min and 20 µL at 25 ± 2°C. pH 3 phosphate buffer solution of the mobile phase was prepared by dissolving 8 g of KH_2_PO_4_ in 100 mL of water and volume was made up to 1000 mL with water, followed by adjusting the solution to pH 3 using dilute H_3_PO_4_. The buffer was filtered using 0.45µm membrane filter (Sigma-Aldrich, Germany) [23].

In order to run the drug content test, a phosphate buffer solution (PBS) of pH 6.8 was prepared using phosphate buffer tablets of pH 6.8 One tablet was dissolved in 100 mL of distilled water with the help of a magnetic stirrer. Each film was placed inside a volumetric flask and 10 mL of PBS was added. The flask was kept aside for 5 min followed by 1-minute mixing using the vortex mixer (Jeiotech VM-96B, Korea) to ensure complete film dissolving. Entrapped air bubbles were removed with the help of the ultrasonic bath for 20 minutes. The solution was filtered through 0.45µm filter and 1 mL of each film solution was taken for HPLC analysis [17, 24]. The test was conducted 3 times for each film formulation and 3 samples of each film solution were taken. Each film was claimed to contain 5 mg of DSL and drug content % was calculated using the following equation [21]. RSD was calculated.


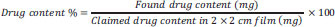


#### 2.2.8 Moisture Loss and Moisture Uptake

Moisture loss test was performed by recording the weights of the films before and after keeping them wrapped in aluminum foil inside the desiccator at room temperature for 72 hours (n=3). Moisture loss % was calculated as % weight loss using the following equation [25].


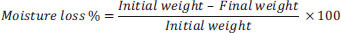


Moisture uptake test was performed by recording the weights of the films before and after keeping them wrapped in aluminum foil inside the humidity chamber (İsolab, Turkey) at room temperature under 75% relative humidity conditions for 72 hours (n=3). Moisture uptake % was calculated as % weight gain using the following equation [26].


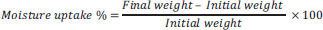


#### 2.2.9 Differential Scanning Calorimetry

The DSC analysis was conducted by analyzing pure DSL, DSL+HPMC physical mixture, and a small part of each film using DSC device (Shimadzu DSC-60, Japan) [27]. Each sample was placed in an aluminum cell that was closed with pressure. The thermal analysis was conducted at a temperature range of 25 to 250°C with a temperature increase of 10°C/min. An empty aluminum sample cell was used as a reference.

#### 2.2.10 Fourier Transform Infrared Spectroscopy

FT-IR test was conducted by analyzing pure DSL, DSL+HPMC physical mixture, and a sample of drug-loaded film using FT-IR device (Shimadzu, Japan) [28]. The test was performed by scanning samples at the wavelength range of 4000-400 cm^-1^ and FT-IR spectra were recorded.

#### 2.2.11 Dissolution Test

Basket apparatus (Pharma test, Germany) was used to carry out the dissolution test. The films were placed inside the baskets and the vessels were filled with 500 mL of PBS (pH 6.8). The test was carried out at 37.5 ± 1°C with the speed of 50 rpm [29]. Samples of 1 mL were collected at time intervals of 15 sec, 30 sec, 1 min, 2 min, 4 min, 8 min, 16 min and 30 min. 1 mL prefilled syringes of blank solution were prepared to replace the taken sample in order to maintain the sink-conditions after each sampling. The samples were filtered using 0.45 µm filter and analyzed for the drug concentration using HPLC analysis. % Cumulative drug release was calculated. For the evaluation of release kinetics, data obtained in the *in vitro* release studies was further investigated for release kinetics using the DDSolver software program [30].

#### 2.2.12 Statistical Analysis

Data were analyzed using SPSS software (Version 20, SPSS Inc., Chicago, IL). The data were expressed as mean ± standard error of the means. To compare the study groups, one-way analysis of variance (ANOVA) was used, followed by Tukey’s post hoc for pairwise comparison. Statistical significance was evaluated at a 0.05 probability level [31].

## 3 RESULTS AND DISCUSSION

### 3.1 Initial Selection of the Optimal Formulation

Selecting the best formulas among all the formulations prepared was the most challenging step during this study. A film of good properties should be smooth with no visible roughness and transparent in appearance [32, 33]. Sticky films are unsuitable for intended application [34]. Physical appearance and texture analysis results are presented in Table **2**. We aimed to get smooth, transparent, non-sticky, and rapidly dissolved film with good mechanical properties. Accordingly, A3, B3, and C3 formulations were excluded from any further studies for not forming a film at all. A6, B6, and C6 formulations were also excluded from any further studies because of the stickiness of formed films that makes them hard to handle. A3, B3, C3, A6, B6, and C6 contain PG as a plasticizer and this indicates its poor plasticizing properties compared to equal amounts of PEG 400 and Gly.

### 3.2 Characterization of ODFs

No specified requirements for the evaluation process of these films are explained in the pharmacopeias due to the newness of this dosage form. The only mentioned requirements in the Ph. Eur. have good mechanical properties to endure handling without breakage and fast disintegration of the films [35]. Optimum ODFs were reported to have low disintegration time with a transparent appearance [2]. Among the selected formulation based on the previous section, transparent with no visible roughness films were subjected to the characterization tests in our study. Other films, especially those near the edges or with visible roughness or imperfections, were excluded from any study. Films succeeded to pass the requirements of disintegration time and mechanical properties were subjected to the rest of the characterization tests.

#### 3.2.1 Disintegration Time

Many disintegration test methods are reported in the literature such as Petri dish method and slide frame method. The volume of the fluid used in the Petri dish method is varied in literature; 2, 4, 10, 15, and 25 mL were used to mimic the small volume of the artificial saliva medium. The petri dish could be swirled at defined time intervals to simulate the rinse conditions over the film’s surface in the mouth medium [35]. Formulas A4, A5, B4, B5, C4, and C5 contain the double quantity of HPMC (600 g) recorded disintegration time ranges from 30 to 70 sec compared to formulas A1, A2, B1, B2, C1, C2, D1, and D2 that have 300 mg HPMC with disintegration time ranges from 4 to 13 sec. It’s obvious that increasing the amount of the polymer increases the time needed for a film to disintegrate. Similar conclusion of prolonged disintegration time related to the increase of HPMC concentration has been noted extensively in the literature [36, 37]. Disintegration time limit of 5-30 s for ODTs can be applied to ODFs [20, 38]. Therefore, formulas A4, A5, B4, B5, C4, and C5 were excluded from further studies for not meeting the requirements of rapidly dissolved oral films.

Results of the best disintegration time of C1, C2, D1, and D2 films are presented in Table **3**. Increasing the amount of plasticizer decreases the disintegration time as D formulations that have a higher amount of the plasticizer disintegrate within 8.50 ± 0.58 to 8.75 ± 0.96 sec compared to C formulations that disintegrate within 11.25 ± 0.50 to 13.25 ± 0.50 sec. Liew *et al*. reported shorter disintegration time when higher concentrations of PEG 400 and Gly concentrations were used [36]. However, any decrease or increase in the plasticizer concentration should be done carefully. High amounts form sticky films and low amounts form films with poor flexibility, as reported in the literature [19]. In our study, films of PEG 400 as plasticizer tend to disintegrate slightly faster than those of Gly films in contrary to what has been reported by Galgatte *et al*. [32] and Kulkarni *et al*. [2]. However, disintegration time values of both C and D formulations were within the accepted range (*p* ˃ 0.05).

#### 3.2.2 Mechanical Properties Evaluation

The mechanical properties of the films are very significant among their characteristics to be taken into consideration when the plan of film preparation is designed; the folding endurance test is the most proper indicator for the actual strength, ease of handling and other mechanical characteristics of the film during the manufacturing and dose administration. High folding endurance value (Table **3**) indicates good mechanical properties. Despite the fact that there is no specific range of folding endurance determined for good mechanical characteristics yet, a value of more than 100 was believed to be good enough for handling [39]. A value of 300 times is sometimes reported as the maximum limit to the test [40]. In our study, a film with a folding endurance value ≥ 100 was considered to owe good mechanical properties. A1, A2, B1, B2 films were already easy to break and hard to handle which led to exclude them from this test and from any further study. C2 and D2 films were noted to pass this test successfully. D1 films could be considered to owe good mechanical characteristics as their endurance values were very close to 100. It’s obvious that increasing the amount of the plasticizer resulted in increasing the folding endurance value and therefore improving the mechanical properties of the film. Significant differences in folding endurance values were reported when equal amounts of PEG 400 and Gly were used. Similar results were obtained by Galgatte *et al*., wherein equal amounts of Gly and PEG 400 provided films with folding endurance values equal to 275 and 135, respectively [32].

#### 3.2.3 Surface pH

In literature, surface pH values of ODFs were mostly in the range of 6.4-6.8 to avoid any irritation to the oral mucosa that causes the patient to spit out the dosage form [25, 41]. Some studies recorded lower values, such as 4.7-5.9 [42], while others recorded higher values up to 7.1 [43]. All our films recorded surface pH values between 6.42 ± 0.03 to 6.60 ± 0.03 (Table **3**), which were within the normal range of oral cavity’s pH. This ensures the absence of any significant change of the pH after the film comes into contact with the oral mucosa and film’s suitability for oral consumption.

#### 3.2.4 Weight Variation

Low weight variation is required to ensure the uniformity of the drug content in the films. A large variation might indicate the inefficiency of the method employed [44]. It was stated that the SD of average % weight variation should not be more than ±7.5% and the % weight variation should not be more than ±15% for an individual film [45, 46]. Accordingly, there was no weight variation reported in our films as the SD values of average % weight variation were lower than 7.5% and the weight of each individual film didn’t deviate more than 15% (Table **4**). It’s unlikely that drug content uniformity could be affected by the weights of the films.

#### 3.2.5 Thickness

Measuring the thickness of the film is fundamental to ensure the uniformity of film thickness as it is one of the major factors that affect the dose of the drug in the film; thus, the drug content uniformity [44]. We measured the thickness at 5 locations of the film including the center point, as many other researchers have done [47, 48], measuring thickness using 3 or 6 different locations was also reported in the literature [20, 24, 49]. The mean value of thickness of all our films ranges between 50.67 ± 1.03 to 53.33 ± 1.03 µm, as shown in Table **5**. In literature, lower values range between 37 ± 81.00 to 53 ± 90.00 µm were reported by Sharma & Agarwal, 2021 [50], while in others, higher values were obtained; 81 ± 1.00 to 96 ± 11.00 μm by Vuddanda *et al*. [49] and 69 to 72 µm by Garcia *et al*. [42]. Generally, it was suggested that an oral film should have a thickness of 50 to1000 µm while others suggested a range of 5 to 200 µm [43]. As our films’ thickness values were within both suggested ranges, they were considered to have a suitable thickness. Low SD values indicate no significant thickness variation. No effect on drug content uniformity is expected to result from thickness variation.

#### 3.2.6 Scanning Electron Microscopy

SEM images were used to evaluate the surface morphology of the films. Absence of pores and surface uniformity is believed to represent a film with good quality [50]. The upper side of initially excluded films have a lot of pores, as shown in Fig. (**1**). Similar SEM images related to films of poor quality have been reported by Alhayali *et al*. [22]. Although we initially excluded such films with visible roughness from any study, we included them in this test just to provide us with a reference of roughness/smoothness when the selected films of the selected formulations are subjected to SEM test.

**Fig. (2):**
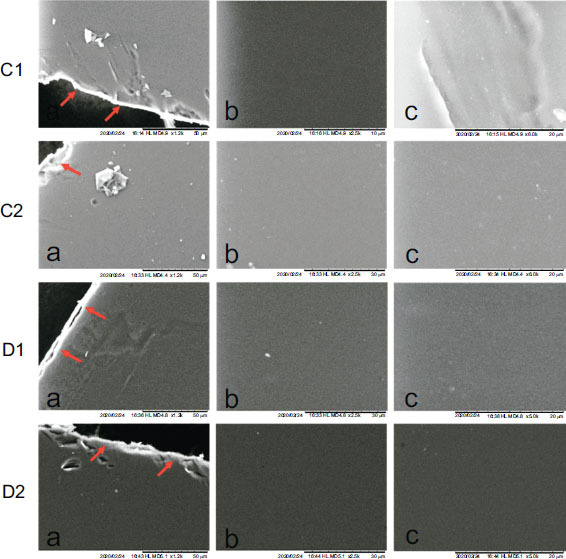
SEM images of C1-D2 films at different magnifications a: 1.2 K X where the red arrows show the edge of the film, b: 2.5 K X, c: 5.0 K X.

The surface morphology of C1, C2, D1, and D2 films showed smooth surfaces with no reported pores. Similar SEM images have been reported in the literature related to films of good quality [22, 51]. This affirmed the smooth texture of the film surface in our formulations and its uniformity and indicated good distribution and complete solubility of the drug particles in the polymeric film matrix (Fig. **2**).

#### 3.2.7 Drug Content and Content Uniformity

Validated HPLC was developed to calculate the drug content. The coefficient of determination (r^2^) for the regression line with the linear equation was 0.9999. Limit of detection and quantitation were 2.7892 and 8.4520 μg/mL, respectively. According to USP 27, the requirements of content uniformity are met if the drug content is within the range of 85-115% of claimed drug content with RSD ≤ 6% [50]. Drug content of C1, D1, and D2 films ranges between 98.3-102.5% with RSD values less than 6% (Table **5**) Accordingly, C1, D1 and D2 formulations met the pharmacopeia requirements of content uniformity. On the other hand, content uniformity of C2 films was not achieved as RSD value was equal to 11.14%. Insufficient content uniformity could lead to batch-to-batch variations. This insufficiency might result from improper viscosity, entrapped air bubbles, or any other problem connected to poor mass spreadability during casting.

#### 3.2.8 Moisture Loss and Moisture Uptake

Moisture loss test’s importance develops from its ability to evaluate a film's capability to maintain its physicochemical properties under normal conditions [45]. Low % moisture loss values in the range of 0.97-1.78% have been reported by Bharti *et al*. and indicated good physical stability and integrity of the film [25]. Relatively higher values have also been reported in the literature; 4.5-6.5% by Reddy & Ramana Murthy [52], 4-6.5% by Reddy *et al.* [26], and 0.66-5.69% by Al-Mogherah *et al.* [27].

In our study, no significant % moisture loss was reported in all formulations. The values were in the range of 0.34-1.99% (Table **3**). This indicates that the films had good integrity and were dry enough to handle after keeping them wrapped in aluminum foil inside the desiccator at room temperature.

The moisture uptake test is important to evaluate the hygroscopic properties of the film [27]. In literature, % moisture uptake ranges of 1.06-9.5% have been reported when films have been kept under the same conditions we applied [26, 52, 53]. C1 and D1 films that contain PEG 400 as a plasticizer showed no significant weight gain according to their very low % moisture uptake values, which were in the range of 0.23-0.98%. Unexpectedly, C2 and D2 films which contain Gly as a plasticizer showed negative % moisture uptake values (Table **3**). It is expected that C2 and D2 films had higher hygroscopic properties as their films absorbed moisture to a point at which their surface started to dissolve since a part of the film was noticed to be stuck to the aluminum foil, as shown in Fig. (**3**). It has been reported that increasing Gly concentration led to an increase in the moisture uptake by the film [17, 27]. Decreasing the plasticizer amount might be considered to solve this problem. Moreover, proper packaging is suggested to protect the films from humidity and to maintain their physicochemical properties.

As a result of the characterization studies on the formulations, there is no significant difference (*p* ˃ 0.05) occurred between C and D formulations, according to lower amounts of plasticizers (C1:glycerin (Gly), C2:polyethylene glycol 400 (PEG 400)), C1 and C2 formulations were chosen for further studies.

#### 3.2.9 Differential Scanning Calorimetry

**Fig. (4):**
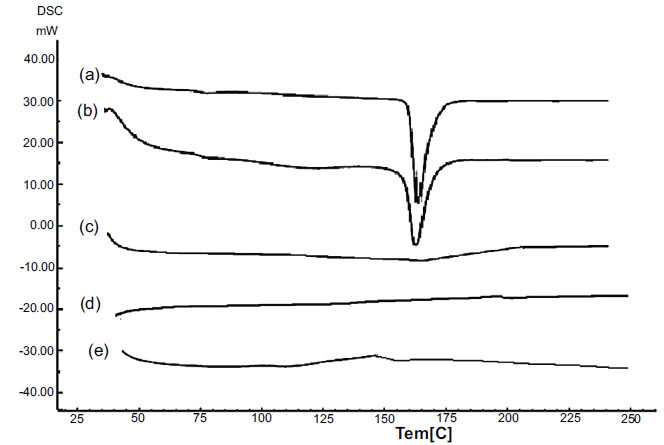
DSC thermograms of: a: DSL, b: DSL+HPMC, c: HPMC, d: C1, e: C2.

DSC test was carried out to investigate the compatibility of the pure substances (drug and polymer) and any possible interactions between the components after the film formation. DSC shows any changes in the enthalpies of a reaction in the shape of the shift of melting endothermic or exothermic peaks and/or variations in them [33]. In our study, DSL thermogram exhibited an endothermic peak corresponding to its melting point at ≈ 160°C (Fig. **4a**). Similar endothermic peak at the same temperature was obtained when a sample of DSL+HPMC was analyzed (Fig. **4b**), which indicates that no physical or chemical interaction has occurred between DSL and the polymer used and proved their compatibility with each other. A decrease in the melting point and the intensity of the peak of the drug when the film is formed have been reported by Raghavendra & Kumar. They also indicated the transformation of the drug state from crystalline to amorphous state because of the dissolution of the drug in the carrier agent at a temperature below its melting point [41]. However, the complete disappearance of the drug peak after film formation has also been reported in the literature. The studies concluded that this loss of the peak might be an indication of the homogenous dispersion of the drug in the film and its presence in an amorphous state [27, 54]. In our study, the characteristic peak of pure DSL disappeared completely in drug loaded films; C1, D1 films (Figs. **4c** - **e**). This indicates that DSL is uniformly dispersed and present in an amorphous state in the polymeric matrix with no interactions with the other excipients.

#### 3.2.10 Fourier Transform Infrared Spectroscopy

FT-IR test was carried out to investigate any possible interactions between the pure drug, the polymer, and the other components after film formation. The FT-IR spectra are compared with each other to detect any changes in the drug spectrum in terms of variation in its characteristic peaks, new peaks, or loss of any peak [45].

**Fig. (5):**
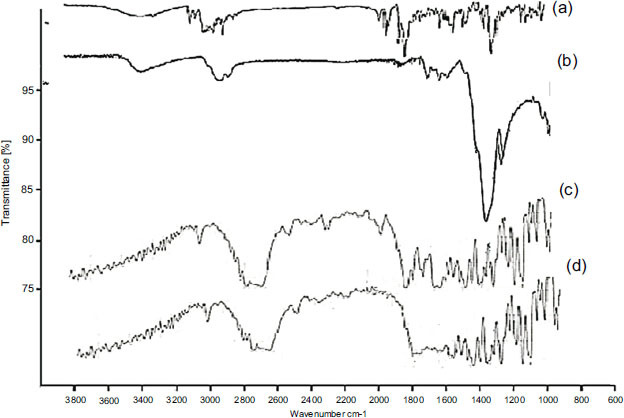
FT-IR spectra of: a: DSL, b: DSL + HPMC, c: C1 d.C2.

The spectra of the physical mixture of DSL+ HPMC and DSL loaded film (Fig. **5**) exhibited the characteristic peaks of DSL spectrum indicating the compatibility of DSL with polymer used. The spectra of DSL show prominent bands at 1.141 and 1.435 cm^-1^, which is attributed to the C-C stretching of aromatic rings, while the bands at 1.172 and 778 cm^-1^ correspond to C-N amines and C-Cl stretching, respectively and is given in Fig. (**5**). The spectra of DSL loaded strip formulations show similar absorption bands, indicating that the DSL did not decompose in the temperatures and was successfully loaded to polymer [55]. The spectra also showed the homogenous dispersion of the drug in the film and also, DSC and FTIR analysis results support each other.

#### 3.2.11 Dissolution Test

Although *in vitro* dissolution test is one of the most frequent tests in the pharmaceutical production to evaluate the drug release profile, no standard dissolution test has been approved to use for the ODFs in any of the available pharmacopeias or regulatory bodies worldwide up to now. As these films are considered solid dosage forms, the available standard dissolution tests of the oral solid dosage forms such as basket apparatus (USP 1), paddle apparatus (USP 2) and the flow-through cell (USP 4) have been used extensively in the literature. However, these methods have many drawbacks resulting in incorrect drug release profiles for oral films [29, 56]. USP 1 was used in our study to carry out the dissolution test. Drug release profiles of C1 and C2 films are shown in Fig. (**6**). Since ODF is a fast disintegrating dosage form, its complete release of the drug will be within minutes. Accordingly, the drug release at 4 minutes was considered to be a measurement for the analysis. Low drug release values in the range of 23-67% have been reported in literature where DSL films were prepared for pediatric use. Gly containing films were reported to dissolute faster than those of PEG 400 [17]. Higher values were reported in our formulations. About 60% of the drug was released after just 15 seconds, while more than 87% of the drug was released by the 4^th^ minute in our formulations. No significant difference was reported between Gly and PEG 400 at the 4^th^ minute. However, Gly containing films exhibited higher drug release values at the 8^th^ minute and at the end of the period time. Despite the original goal and preparations to maintain the sink- conditions, our data was collected under non-sink conditions. It was hard to replace the taken sample with an equal blank solution since the sampling time intervals are very small especially in the first-minute interval that has 3 sampling points (15, 30 and 60 sec). The different pharmacopeias (Ph. Eur. and USP) suggest the use of sink-condi-tions to run a dissolution test. However, when a medium fails to provide sink conditions, non-sink conditions may be acceptable if supported by a suitable justification [29]. In fact, there are few studies that carried out the dissolution test of oral films under non-sink conditions [49]. Accordingly, we didn’t run the dissolution test for the rest of the formulations (D1 and D2). We believe that conventional USP dissolution apparatuses are unable to characterize the drug release profile of oral films since they don’t simulate the physiology of the oral cavity, such as the small saliva volume, saliva flow rate and the force applied by the tongue. Customized methods are needed to mimic these conditions. Moreover, automatic rather than manual sampling is suggested since the drug release is a matter of seconds in these films. This requires complicated instrumental setup and complex manual operations, which prevented us from running the standard test using USP 1 apparatus for the rest of the formulations.

*In vitro* release rate analyses were evaluated for the determination of DSL release models from the strips by DDsolver program and the R^2^ and R^2^_adjusted_ values for the Zero-order, First-order, Higuchi, Korsmeyer-Peppas, Hixson-Crowell, Hopfenberg, Baker-Lonsdale and Peppas-Sahlin are smaller than Weibull model for E2 formulation, the drug release does not comply with these models. The Weibull model was found to be more suitable than the other release kinetics. Also the value of the exponent ‘β’ is a parameter of the mechanism of transport of a drug through the polymeric matrix. The ‘β’ value of the C1 and C2 strips prepared in this study was found to be 0.893 and 0.852 showing that the active substance from strips was released according to fickian diffusion (*p* ˃ 0.05) [30, 57].

## CONCLUSION

Orodispersible films are novel dosage forms that have been a subject of interest in the previous years. HPMC was the best film forming agent among the used polymers (HPMC, PVA, and Eudragit RS 100) in terms of film forming ability, transparency, and lack of stickiness. A lower concentration of HPMC resulted in lower disintegration time. Increasing the amount of plasticizers has a significant effect on the mechanical properties of the film. Our formulations with a pH within the range of normal pH of the oral cavity and this indicates the suitability of this dosage form and the success of solvent casting method in preparing 5 mg DSL films for oral consumption as an alternative to conventional dosage forms with higher patient compliance and convenience to treat allergic symptoms in geriatric patients.

## Figures and Tables

**Fig. (1):**
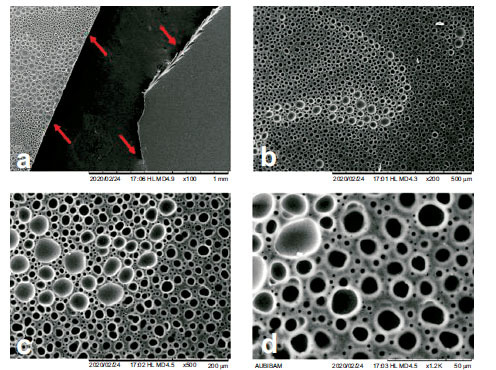
SEM images of initially excluded film at different magnifications as an indicator of film roughness/smoothness, a: 100X where the red arrows show the edges of two samples of the same film; smooth on right and rough on left, b: 200 X, c: 500 X, d: 1.2 K X.

**Fig. (3):**
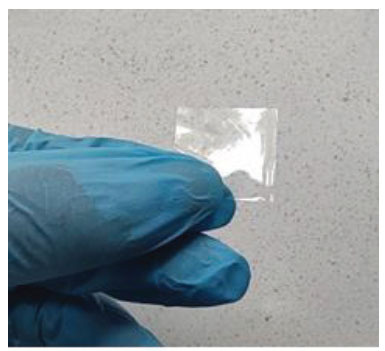
A part of Gly film stuck to the aluminum foil because of moisture uptake.

**Fig. (6):**
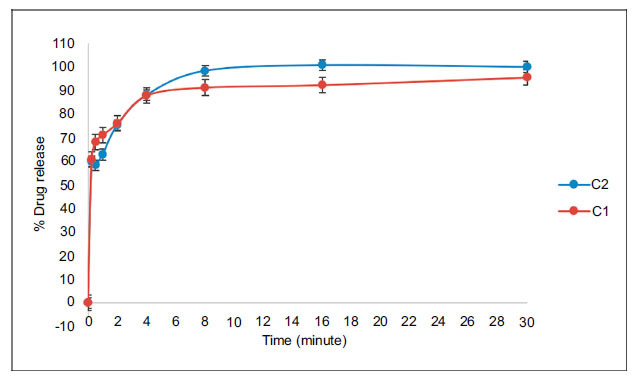
% Drug release profiles of C1 and C2 films.

**Table 1:** The composition of the formulations.

**Formulation Code**	**DSL (mg)**	**HPMC (mg)**	**PEG 400 (mg)**	**Gly (mg)**	**PG (mg)**	**CA (mg)**	**Water (mL)**	**Ethanol (mL)**
A1	88	300	45	-	-	25	3	7
A2	88	300	-	45	-	25	3	7
A3	88	300	-	-	45	25	3	7
A4	88	600	45	-	-	25	3	7
A5	88	600	-	45	-	25	3	7
A6	88	600	-	-	45	25	3	7
B1	88	300	67.5	-	-	25	3	7
B2	88	300	-	67.5	-	25	3	7
B3	88	300	-	-	67.5	25	3	7
B4	88	600	67.5	-	-	25	3	7
B5	88	600	-	67.5	-	25	3	7
B6	88	600	-	-	67.5	25	3	7
C1	88	300	90	-	-	25	3	7
C2	88	300	-	90	-	25	3	7
C3	88	300	-	-	90	25	3	7
C4	88	600	90	-	-	25	3	7
C5	88	600	-	90	-	25	3	7
C6	88	600	-	-	90	25	3	7
D1	88	300	120	-	-	25	3	7
D2	88	300	-	120	-	25	3	7

**Table 2:** Physical appearance and texture analysis of A1-D2 films.

**Formulation Code**	**Film Formation**	**Transparency**	**Stickiness**
A1, A2, A4, A5, B1, B2, B4, B5, C1, C2, C4, C5, D1, D2	Yes	Transparent	Not sticky
A3, B3, C3	No	-	-
A6, B6, C6	Yes	Not transparent	Sticky

**Table 3:** Characterization tests results of C1, C2, D1, and D2 films. Data are presented as mean ± SD.

**Film Code**	**Disintegration Time (sec) (n = 4)**	**Folding endurance (times) (n = 4)**	**Surface pH (n = 3)**	**Thickness (µm) (n = 6)**	**Moisture Loss (%) (n = 3)**	**Moisture Uptake (%) (n = 3)**
C1	11.25 ± 0.50	62.25 ± 4.03	6.42 ± 0.03	50.67 ± 1.03	0.99 ± 0.27	0.69 ± 0.39
C2	13.25 ± 0.50	103.75 ± 5.06	6.59 ± 0.01	51.33 ± 1.03	1.99 ± 0.35	-1.80 ± 0.64
D1	8.50 ± 0.58	96.25 ± 2.99	6.48 ± 0.02	53.33 ± 1.03	0.34 ± 0.12	0.61 ± 0.33
D2	8.75 ± 0.96	311.25 ± 11.09	6.60 ± 0.03	52.67± 1.03	1.66 ± 0.11	-0.94 ± 0.38

**Table 4:** Weight and % weight variation of C1, C2, D1, and D2 films.

**Film Code**	**Weight (mg ± SD)**	**Weight Deviation (%)**
C1	18.13 ± 1.13	6.25
C2	17.91 ± 0.53	2.99
D1	20.02 ± 1.39	6.93
D2	19.10 ± 0.83	4.34

**Table 5:** Drug content of C1, C2, D1, and D2 films.

**Film Code**	**Drug Content (mg ± SD)**	**Drug Content (% ± RSD)**
C1	4.92 ± 0.18	98.30 ± 3.56
C2	5.22 ± 0.58	104.46 ± 11.14
D1	5.12 ± 0.19	102.49 ± 3.76
D2	5.09 ± 0.13	101.82 ± 2.56

## Data Availability

The data that support the findings of this study are available from the corresponding author, [EY], upon reasonable request.

## LIST OF ABBREVIATIONS

CA: Citric Acid
DSC: Differential Scanning Calorimetry
DSL: Desloratadine
EMA: European Medicines Agency
FDA: Food and Drug Administration
FT-IR: Fourier Transform Infrared Spectroscopy
Gly: Glycerol
HPLC: High Pressure Liquid Chromatography
HPMC: Hydroxypropyl Methylcellulose
ODF: Orodispersible Film
ODTs: Disintegrating Tablets
PBS: Phosphate Buffer Solution
PEG 400: Polyethylene Glycol 400
SEM: Scanning Electron Microscope

## References

[1] Darji M.A., Lalge R.M., Marathe S.P., Mulay T.D., Fatima T., Alshammari A., Lee H.K., Repka M.A., Narasimha Murthy S. (2018). Excipient stability in oral solid dosage forms: A review.. AAPS PharmSciTech.

[2] Kulkarni A.S., Deokule H.A., Mane M.S., Ghadge D.M. (2010). Exploration of different polymers for use in the formulation of oral fast dissolving strips.. J. Curr. Pharm. Res. JCPR..

[3] Stegemann S., Ecker F., Maio M., Kraahs P., Wohlfart R., Breitkreutz J., Zimmer A., Bar-Shalom D., Hettrich P., Broegmann B. (2010). Geriatric drug therapy: Neglecting the inevitable majority.. Ageing Res. Rev..

[4] Wahlich J., Stegemann S., Orlu-Gul M. (2013). Meeting commentary—“Medicines for older adults: Learning from practice to develop patient centric drug products”.. Int. J. Pharm..

[5] Okuda Y., Irisawa Y., Okimoto K., Osawa T., Yamashita S. (2009). A new formulation for orally disintegrating tablets using a suspension spray-coating method.. Int. J. Pharm..

[6] Gryczke A., Schminke S., Maniruzzaman M., Beck J., Douroumis D. (2011). Development and evaluation of orally disintegrating tablets (ODTs) containing Ibuprofen granules prepared by hot melt extrusion.. Colloids Surf. B Biointerfaces.

[7] Dixit R.P., Puthli S.P. (2009). Oral strip technology: Overview and future potential.. J. Control. Release.

[8] Al-khattawi A., Mohammed A.R. (2013). Compressed orally disintegrating tablets: Excipients evolution and formulation strategies.. Expert Opin. Drug Deliv..

[9] Liew K.B., Tan Y.T.F., Peh K.K. (2012). Characterization of oral disintegrating film containing donepezil for Alzheimer disease.. AAPS PharmSciTech.

[10] Patel J.G., Modi A.D. (2012). Formulation, optimization and evaluation of levocetirizine dihyrochloride oral thin strip.. J. Pharm. Bioallied Sci..

[11] Irfan M., Rabel S., Bukhtar Q., Qadir M.I., Jabeen F., Khan A. (2016). Orally disintegrating films: A modern expansion in drug delivery system.. Saudi Pharm. J..

[12] Karki S., Kim H., Na S.J., Shin D., Jo K., Lee J. (2016). Thin films as an emerging platform for drug delivery.. Asian J. Pharmaceut. Sci..

[13] Gholve S., Savalsure S., Bhusnure O., Surywanshi S., Birajdar M. (2018). Formulation and evaluation of oral fast dissolving sublingual film of formulation and evaluation of oral fast dissolving sublingual film of propranolol HCl.. Int. J. Pharma Res. Heal. Sci..

[14] Borges A.F., Silva C., Coelho J.F.J., Simões S. (2015). Oral films: Current status and future perspectives.. J. Control. Release.

[15] Łagun A. (2017). Desloratadine in the treatment of allergic rhinitis and urticaria in a daily practice of family doctors.. Lek. POZ..

[16] Buczak K., Sybilski A.J. (2018). The role of desloratadine in the treatment of allergic rhinitis and urticaria.. Lek. POZ..

[17] Singh H., Kaur M., Verma H. (2013). Optimization and evaluation of desloratadine oral strip: An innovation in paediatric medication.. ScientificWorldJournal.

[18] Joshua J.M., Hari R., Jyothish F.K., Surendran S.A. (2016). Fast dissolving oral thin films: An effective dosage form for quick releases.. Int. J. Pharm. Sci. Rev. Res..

[19] Chaudhary H., Gauri S., Rathee P., Kumar V. (2013). Development and optimization of fast dissolving oro-dispersible films of granisetron HCl using Box–Behnken statistical design.. Bull. Fac. Pharm. Cairo Univ..

[20] Rençber S., Karavana S.Y., Yilmaz F.F., Eraç B., Nenni M., Gurer-Orhan H., Limoncu M.H., Güneri P., Ertan G. (2019). Formulation and evaluation of fluconazole loaded oral strips for local treatment of oral candidiasis.. J. Drug Deliv. Sci. Technol..

[21] Centkowska K., Ławrecka E., Sznitowska M. (2020). Technology of orodispersible polymer films with micronized loratadine—influence of different drug loadings on film properties.. Pharmaceutics.

[22] Alhayali A., Vuddanda P.R., Velaga S. (2019). Silodosin oral films: Development, physico-mechanical properties and in vitro dissolution studies in simulated saliva.. J. Drug Deliv. Sci. Technol..

[23] Bondili S., Ramya M. (2011). Method development and validation of desloratadine in bulk and its tablet dosage forms.. Int. J. Pharm. Ind. Res..

[24] Patil A.B., Charyulu R.N., Shastry C.S. (2013). Development and characterization of atenolol fast dissolving orodispersible films.. World J. Pharm. Res..

[25] Bharti K., Mittal P., Mishra B. (2019). Formulation and characterization of fast dissolving oral films containing buspirone hydrochloride nanoparticles using design of experiment.. J. Drug Deliv. Sci. Technol..

[26] Sudhakara Reddy P., Koteswara Rao G.S.N., Ramana Murthy K.V. (2016). Formulation and evaluation of oral fast dissolving films of poorly soluble drug loperamide HCl using transcutol HP.. Int. J. Adv. Pharma. Biotechnol..

[27] Al-Mogherah A.I., Ibrahim M.A., Hassan M.A. (2020). Optimization and evaluation of venlafaxine hydrochloride fast dissolving oral films.. Saudi Pharm. J..

[28] Panchal M.S., Patel H., Bagada A., Vadalia K.R. (2012). Formulation and evaluation of mouth dissolving film of ropinirole hydrochloride by using pullulan polymers.. Int. J. Pharm. Res. Allied Sci..

[29] Adrover A., Pedacchia A., Petralito S., Spera R. (2015). in vitro dissolution testing of oral thin films: A comparison between USP 1, USP 2 apparatuses and a new millifluidic flow-through device.. Chem. Eng. Res. Des..

[30] Yenilmez E., Öztürk A.A., Başaran E. (2023). Preparation and in vitro, ex vivo evaluation of benzidamine hydrochloride loaded fast dissolving oral strip formulations: Treatment of oral mucositis due to side effects of chemotherapy and radiotherapy.. Lett. Drug Des. Discov..

[31] Ullah W., Nawaz A., Akhlaq M., Shah K.U., Latif M.S., Doolaanea A.A., Alfatama M. (2021). Transdermal delivery of gatifloxacin carboxymethyl cellulose-based patches: Preparation and characterization.. J. Drug Deliv. Sci. Technol..

[32] Galgatte U.C., Khanchandani S.S., Jadhav Y.G., Chaudhari P.D. (2013). Investigation of different polymers, plasticizers and superdisintegrating agents alone and in combination for use in the formulation of fast dissolving oral films.. Int. J. Pharm. Tech. Res..

[33] Abdelbary A., Bendas E.R., Ramadan A.A., Mostafa D.A. (2014). Pharmaceutical and pharmacokinetic evaluation of a novel fast dissolving film formulation of flupentixol dihydrochloride.. AAPS PharmSciTech.

[34] Garsuch V., Breitkreutz J. (2010). Comparative investigations on different polymers for the preparation of fast-dissolving oral films.. J. Pharm. Pharmacol..

[35] Speer I., Steiner D., Thabet Y., Breitkreutz J., Kwade A. (2018). Comparative study on disintegration methods for oral film preparations.. Eur. J. Pharm. Biopharm..

[36] Liew K.B., Tan Y.T.F., Peh K.K. (2014). Effect of polymer, plasticizer and filler on orally disintegrating film.. Drug Dev. Ind. Pharm..

[37] Raju P.N., Kumar M.S., Reddy C.M., Ravishankar K. (2013). Formulation and evaluation of fast dissolving films of loratidine by solvent casting method.. Pharma Innov..

[38] Bhyan B., Jangra S., Kaur M., Singh H. (2011). Orally fast dissolving films: Innovations in formulation and technology.. Int. J. Pharm. Sci. Rev. Res..

[39] Takeuchi Y., Ikeda N., Tahara K., Takeuchi H. (2020). Mechanical characteristics of orally disintegrating films: Comparison of folding endurance and tensile properties.. Int. J. Pharm..

[40] Morales J.O., McConville J.T. (2011). Manufacture and characterization of mucoadhesive buccal films.. Eur. J. Pharm. Biopharm..

[41] Hl R., Kumar G.P. (2016). Development and evaluation of polymer-bound glibenclamide oral thin film.. J. Bioequivalence Bioavailab..

[42] Garcia V.A.S., Borges J.G., Maciel V.B.V., Mazalli M.R., Lapa-Guimaraes J.G., Vanin F.M., de Carvalho R.A. (2018). Gelatin/starch orally disintegrating films as a promising system for vitamin C delivery.. Food Hydrocoll..

[43] Sjöholm E., Sandler N. (2019). Additive manufacturing of personalized orodispersible warfarin films.. Int. J. Pharm..

[44] Nair A.B., Kumria R., Harsha S., Attimarad M., Al-Dhubiab B.E., Alhaider I.A. (2013). In vitro techniques to evaluate buccal films.. J. Control. Release.

[45] Dharmasthala S., Shabaraya A.R., Andrade G.S., Shriram R.G., Hebbar S., Dubey A. (2018). Fast dissolving oral film of piroxicam: Solubility enhancement by forming an inclusion complex with β-cyclodextrin, formulation and evaluation.. J. Young Pharm..

[46] Mushtaque M., Muhammad I.N., Fareed Hassan S.M., Ali A., Masood R. (2020). Development and pharmaceutical evaluation of oral fast dissolving thin film of escitalopram: A patient friendly dosage form.. Pak. J. Pharm. Sci..

[47] Thakur R.R., Rathore D.S., Narwal S. (2012). Orally disintegrating preparations: Recent advancement in formulation and technology.. J. Drug Deliv. Ther..

[48] Castro P.M., Sousa F., Magalhães R., Ruiz-Henestrosa V.M.P., Pilosof A.M.R., Madureira A.R., Sarmento B., Pintado M.E. (2018). Incorporation of beads into oral films for buccal and oral delivery of bioactive molecules.. Carbohydr. Polym..

[49] Vuddanda P.R., Montenegro-Nicolini M., Morales J.O., Velaga S. (2017). Effect of surfactants and drug load on physico-mechanical and dissolution properties of nanocrystalline tadalafil-loaded oral films.. Eur. J. Pharm. Sci..

[50] Sharma A., Agarwal D. (2021). Formulation and evaluation of montelukast sodium oral dissolving film.. Asian J. Pharmaceut. Res. Develop..

[51] Pimparade M.B., Vo A., Maurya A.S., Bae J., Morott J.T., Feng X., Kim D.W., Kulkarni V.I., Tiwari R., Vanaja K., Murthy R., Shivakumar H.N., Neupane D., Mishra S.R., Murthy S.N., Repka M.A. (2017). Development and evaluation of an oral fast disintegrating anti-allergic film using hot-melt extrusion technology.. Eur. J. Pharm. Biopharm..

[52] Reddy P.S., Murthy K.V.R. (2018). Formulation and evaluation of oral fast dissolving films of poorly soluble drug ezetimibe using transcutol Hp, Indian J.. Indian J. Pharmaceut. Edu. Res..

[53] Sheikh F.A., Aamir M.N., Shah M.A., Ali L., Anwer K., Javaid Z. (2020). Formulation design, characterization and in vitro drug release study of orodispersible film comprising BCS class II drugs.. Pak. J. Pharm. Sci..

[54] Bala R., Khanna S., Pawar P. (2014). Design optimization and in vitro-in vivo evaluation of orally dissolving strips of clobazam.. J. Drug Deliv..

[55] Yenılmez E. (2017). Desloratadine-Eudragit® RS100 nanoparticles: formulation and characterization.. Turkish J. Pharmaceut. Sci..

[56] Speer I., Preis M., Breitkreutz J. (2019). Dissolution testing of oral film preparations: Experimental comparison of compendial and non-compendial methods.. Int. J. Pharm..

[57] Öztürk A.A., Kıyan H.T. (2020). Treatment of oxidative stress-induced pain and inflammation with dexketoprofen trometamol loaded different molecular weight chitosan nanoparticles: Formulation, characterization and anti-inflammatory activity by using in vivo HET-CAM assay.. Microvasc. Res..

